# Acid-etched monolithic translucent zirconia laminate veneer for restoring a discolored anterior tooth: A case report

**DOI:** 10.1097/MD.0000000000042093

**Published:** 2025-04-04

**Authors:** Nguyen Viet Anh, Nguyen Thi Trang, Nguyen Thi Loan

**Affiliations:** a Faculty of Dentistry, Phenikaa University, Hanoi, Viet Nam; b Viet Anh Orthodontic Clinic, General Dentistry Department, Hanoi, Vietnam.

**Keywords:** acid-etched zirconia, bond strength, discolored tooth, monolithic zirconia, translucent zirconia

## Abstract

**Rationale:**

Laminate veneers have become the treatment of choice for restoring tooth esthetics and function conservatively. However, due to their thin nature, the esthetic rehabilitation of discolored teeth following pulp necrosis can be challenging. Translucent zirconia offers a favorable balance between masking ability and optical translucency while maintaining high durability. To enhance the bonding strength between zirconia and adhesive agents, the treatment of the zirconia surface with strong acids has been extensively investigated in laboratory settings. However, the clinical application of this technique has not yet been published.

**Patient concerns:**

A discolored anterior incisor that had undergone endodontic treatment.

**Diagnosis:**

Discolored anterior incisor following pulp necrosis.

**Interventions:**

Restoration of the discolored anterior incisor with an acid-etched translucent zirconia veneer.

**Outcomes:**

The monolithic translucent zirconia veneer successfully restored the discolored anterior tooth. A 2-year follow-up demonstrated that the acid-etched surface ensured the long-term survival of the restoration.

**Lessons:**

Enhancing bond strength through micromechanical retention created by strong acid etching has proven effective in ensuring a secure bond between the veneer and adhesive cement, contributing to the long-term success of translucent zirconia veneers.

## 1. Introduction

Since anterior teeth play a crucial role in the esthetic appearance of the face, their restoration is 1 of the most common reasons patients seek treatment in cosmetic dentistry. Following dental pulp necrosis, teeth often experience color changes due to the deposition of blood residues within the dentinal tubules. In the anterior region, endodontically treated discolored teeth can be restored through various approaches, including full dental crowns, non-vital bleaching followed by crowns, or laminate veneers. Compared to full ceramic crowns, the preparation of laminate veneers requires minimal reduction of tooth structure, making them more easily accepted by patients.^[1]^

Among the ceramics used in dentistry, zirconia is 1 of the most popular due to its biocompatibility and physical hardness. Zirconia opacity is sufficient to cover discolored teeth, and its physical properties ensure long-term survival. Historically, zirconia was primarily used for posterior tooth restorations or as the inner frame of veneers or dental crowns due to its physical hardness and opacity appearance.^[2]^ In recent years, the advent of translucent zirconia has expanded its use to anterior teeth. The improved optical properties of this type of zirconia allow for the fabrication of veneers entirely from zirconia, reducing the need for tooth reduction.^[3]^

However, a significant challenge with zirconia restorations is their bonding ability with the current cement systems. The retention of veneers relies solely on the chemical adhesion and micromechanical retention between the resin cement and the intaglio surface of the ceramic veneer. Zirconia is a highly crystalline material, chemically inert to common surface treatments. Thus, it is difficult to obtain retention through micromechanical interlocks and silanation using silica-based primers, as with glass-based ceramics.^[4]^ These factors limit the applications of zirconia for anterior tooth restorations as laminate veneers. Traditionally, zirconia crowns are treated with sand-blasting in the laboratory to enhance bonding strength. However, this procedure may cause microcracks and reduce the durability of the restoration under masticatory load.^[5]^ To overcome this challenge, various zirconia surface treatment methods, both micromechanical and chemical, have been proposed and extensively investigated, including airborne particle abrasion, tribochemical silica coating, vitrification and glazing, laser etching, and selective infiltration etching.^[4]^ Recently, a surface architecting technique for zirconia using a combination of HNO_3_-HF acids has been published, showing promising results in both surface roughness and bonding strength. This acid mixture creates a more micromechanically retentive surface on zirconia and provides higher bonding strength compared to traditional surface abrasion techniques or chemical modification with a layer of glass ceramics.^[6]^ Although the efficacy of this technique has been verified in the laboratory, there are no reports on its application in a clinical setting. This case report presents the successful application of a novel approach using acid etching on the intaglio surface of monolithic translucent zirconia veneers to treat a discolored anterior tooth, with positive results observed after a 2-year follow-up.

## 2. Case presentation

A 27-year-old female patient presented to our clinic with the chief complaint of a dark color left central incisor. The patient reported having that tooth endodontically treated many years ago due to trauma from an accident. The tooth color remains like that for many years.

## 3. Treatment planning

**Figure 1.**
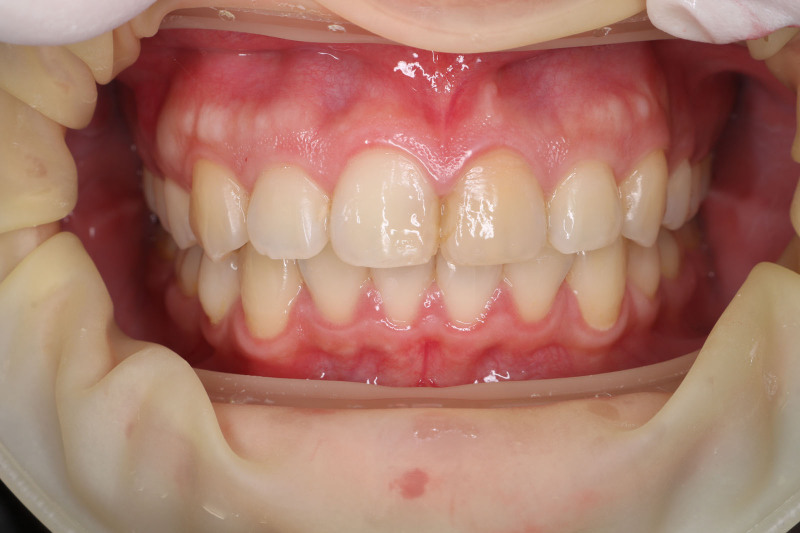
Preoperative photograph. The left central incisor was discolored following trauma many years ago.

**Figure 2.**
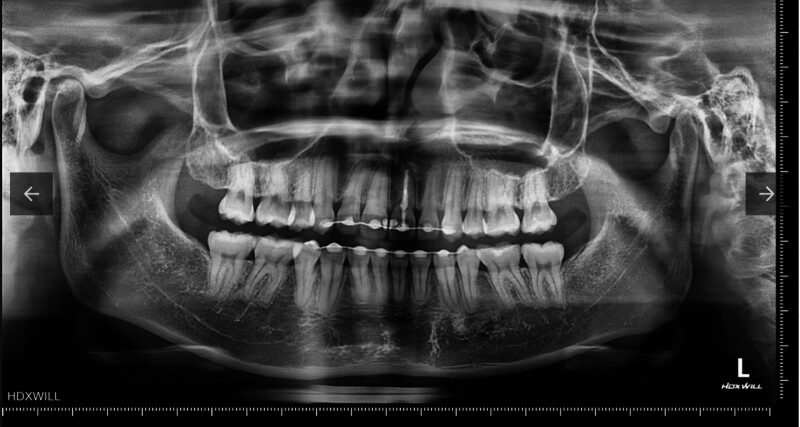
Preoperative radiograph. The left central incisor had been endodontically treated, showing no signs or symptoms of infection.

The patient’s medical history was uneventful, with no history of bruxism or tooth clenching identified. An intraoral examination revealed a discolored left central incisor that had been endodontically treated. There were no noticeable signs or symptoms upon examination, nor were any abnormalities observed on the radiograph (Figs. 1 and 2). To improve the esthetic appearance of the tooth, various treatment options were discussed, including a full ceramic crown and an indirect laminate veneer. The patient chose the conservative ceramic veneer option. Informed consent was obtained before executing the treatment. The patient received dental prophylaxis and oral hygiene instructions. The future prosthesis was evaluated using a study model and a diagnostic mockup made of light-cure composite resin. After careful consideration, a ceramic veneer restoration fabricated from highly translucent zirconia was selected.

## 4. Tooth preparation

**Figure 3.**
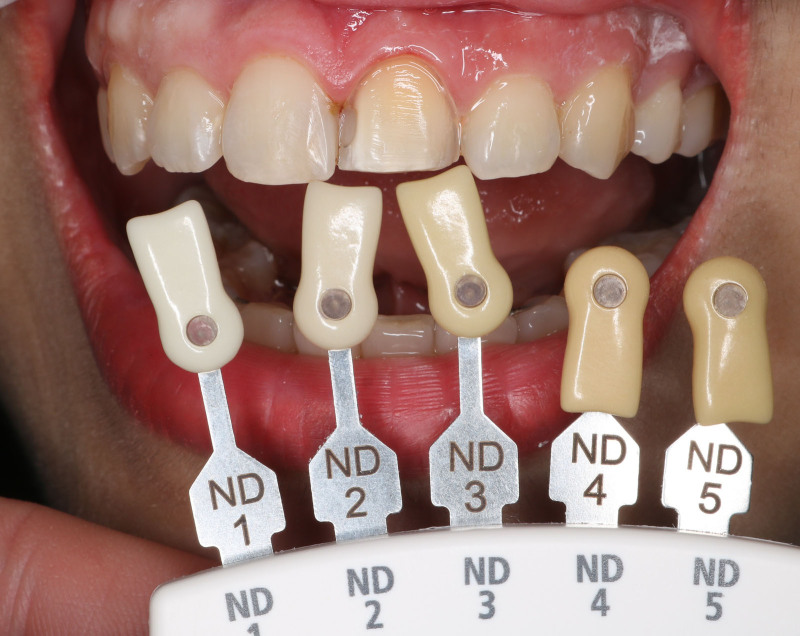
Selecting the shade of the left central incisor abutment.

**Figure 4.**
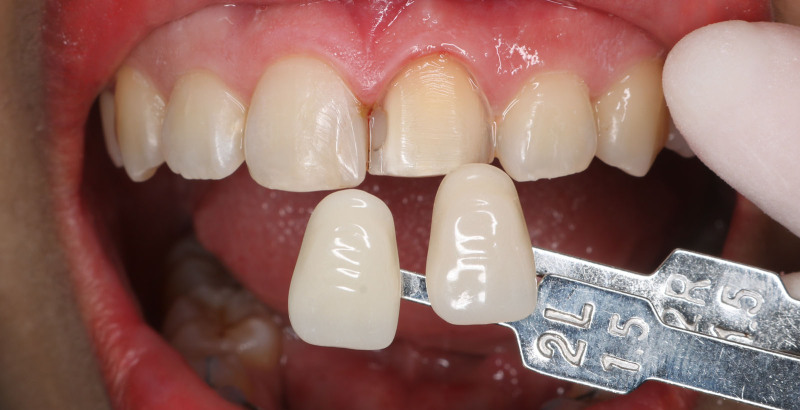
Selecting the shade of the adjacent teeth.

Minimally invasive preparation was performed on the left central incisor. The buccal surface of the tooth was uniformly reduced by 0.5 mm. A 2 mm reduction was attempted at the incisal edge. Care was taken to confine the preparation within the enamel layer. The cervical finishing line was placed slightly under the gingival margin. All the corners were rounded and the preparation was polished with fine diamond burs. The shade of prepared and adjacent teeth was then selected (IPS natural die material shade guide, Ivoclar, Germany) (Figs. 3 and 4).

## 5. Impression

An impression of the prepared tooth was taken using silicone putty on a stock tray with double cords impression technique (Elite P&P, Zhermack, Italy).

## 6. Provisional restoration

After taking the impression, the abutment of the left central incisor was temporarily restored with a light-curing composite resin (3M™ Filtek™ Z350XT Flowable, 3M) to protect the tooth from saliva and provide proper aesthetics.

## 7. Fabrication of the veneer

The master cast was poured using type IV gypsum (Elite Rock, Zhermack, Italy). The stone model was scanned, and the veneer was milled from monolithic translucent zirconia (Nacera® Pearl Q³ Multi-Shade, Dental Direkt GmbH, Germany) using a CAD/CAM system (CORiTEC, Imes-Icore, Germany). Before sintering, a layer of opaque liquid (Prettau® Aquarell, Zirkonzahn, Italy) was applied to the bonding surface of the milled veneer to aid in covering the dark abutment. The intaglio surface of the veneer was then etched with a mixture of zirconia etching acids (ZIRCOS-E, Eunjin Chemical, South Korea) for 2 hours at room temperature, thereafter thoroughly washed with running distilled water. After being fired in a furnace at 1150 °C for 1 hour for annealing, the veneer was glazed and fired at 900 °C for 10 minutes.^[7]^

## 8. Cementation

The procedure was executed in moisture-controlled conditions employing rubber dam isolation. The shape, esthetics, and marginal adaptation of the veneer were verified intraorally. Once everything was satisfactory, the resin cement shade was selected using try-in pastes (RelyX Try-In Paste, 3M). After cleaning and drying the ceramic veneer in an ultrasonic bath for 15 minutes, a universal primer containing 10-MDP and silane (Single Bond Universal, 3M) was applied for 20 seconds and air-dried for 5 seconds to remove all solvents. The selected shade of light-cured veneer cement (RelyX Veneer LC, 3M) was then applied to the bonding surface of the veneer, with gentle drying for 5 seconds.

**Figure 5.**
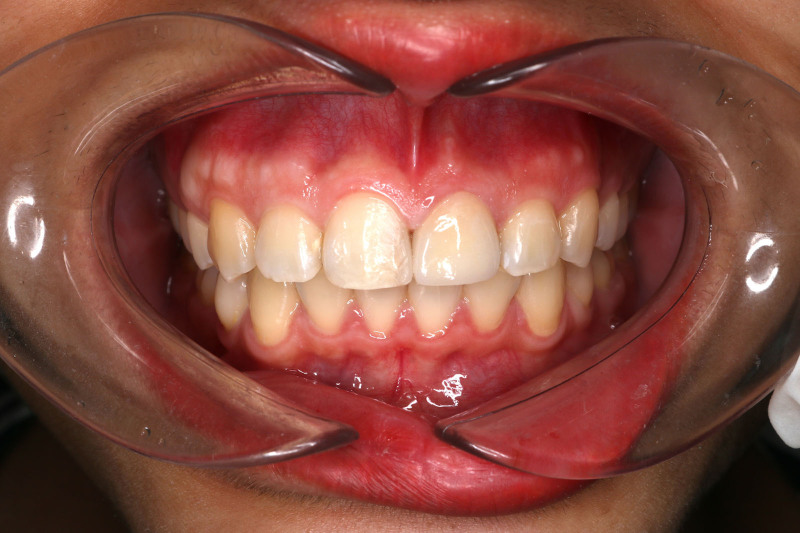
Intraoral photograph of the left central incisor after veneer cementation.

**Figure 6.**
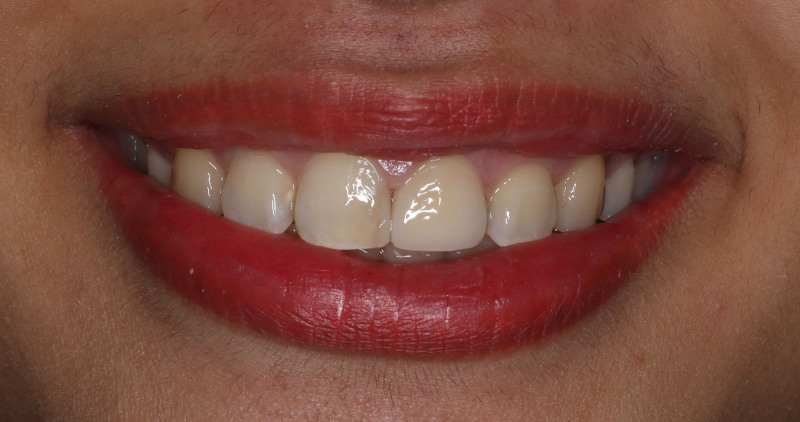
Extraoral photograph of the left central incisor after veneer cementation.

The tooth abutment was prepared with fluoride-free pumice powder and then etched with 37% phosphoric acid (Total Etch, Ivoclar) for 15 seconds. After washing and drying, a bonding agent (Single Bond Universal, 3M) was applied for 20 seconds, gently air-dried for 5 seconds, and light-curing for ten seconds (Woodpecker LED.F, China). The veneer with the adhesive cement was seated on the tooth surface, and excess cement was removed from the margins and interproximal surfaces. Light polymerization (Woodpecker LED.F, China) was then performed for 30 seconds on each margin (gingival, incisal edge, and proximal). After final marginal polishing, occlusion was checked for any disturbances. Postoperative intraoral and extraoral photographs of the patient are shown in Figures 5 and 6, respectively. The patient’s satisfaction was evaluated through an oral interview, and she was very satisfied with the result.

**Figure 7.**
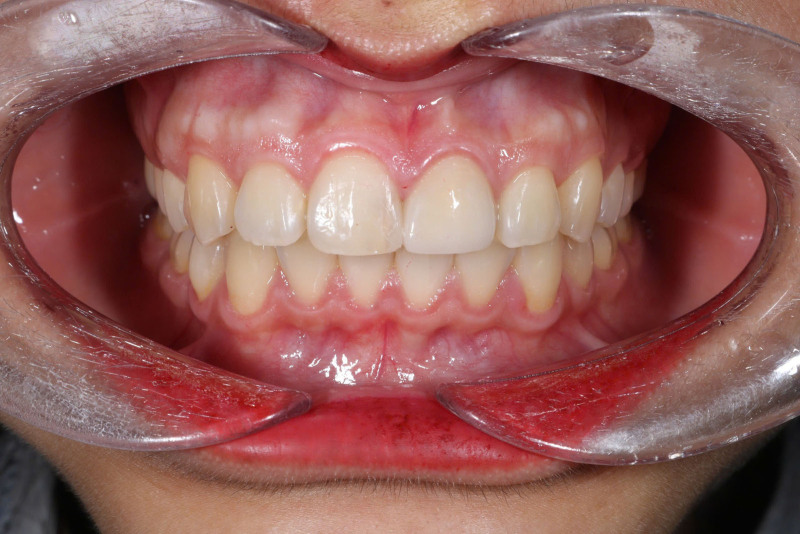
Two-year follow-up intraoral photograph of the left central incisor veneer.

The patient returned for follow-up examinations 1 week, 1 year and 2 years later. No signs of dislodgement or marginal gaps were observed at the 2-year follow-up (Fig. 7).

## 9. Discussion

This case report presented the restoration of a discolored central incisor following pulp necrosis using an acid-etched translucent zirconia veneer. To the best of our knowledge, this is the first case report on the use of this technique for translucent zirconia veneers. The overall esthetic and functional outcomes were satisfactory upon 2-year follow-up.

To restore discolored anterior teeth, the veneer must be opaque enough to mask the underlying dark abutment while maintaining good optical properties for esthetics. Various ceramics have been used for laminate veneer restorations, with lithium disilicate being 1 of the most common choices for anterior teeth. This ceramic contains a specific amount of disilicate crystals within the glassy matrix, providing good control over physical properties. Additionally, high bond strength can be achieved through etching with hydrofluoric acid, and the low refractive index of lithium disilicate crystals allows for natural tooth color restoration.^[8]^ However, due to their high translucency, these ceramic materials may not effectively mask a dark abutment. Another alternative could be utilizing non-vital tooth bleaching before performing veneer restoration. Bleaching can improve the color of the tooth abutment, potentially making the use of lithium disilicate ceramic feasible. However, in this patient, the open apex and the enlargement of the root canal observed on the radiograph, particularly in the cervical region, suggest that the tooth experienced trauma at an early developmental stage. In such cases, the dentinal tubules are often larger than in mature teeth, posing a risk of bleaching agents permeating into the periodontal ligament and causing cervical root resorption.^[9]^ The option of using an opaque zirconia frame under layers of glass ceramic is also possible. However, in veneer restorations, creating space for both the frame and the outer ceramic layer often requires more tooth reduction, which poses a risk of exposing the dentin layer and compromising the bonding strength of the adhesive system.^[10]^

Translucent zirconia was developed from traditional zirconia used in dentistry (3 mol% yttria-stabilized tetragonal zirconia polycrystal) by altering the concentration of yttrium oxide. Increasing the yttria content from 3% to 5% enhances translucency by increasing the cubic phase within zirconia.^[3]^ Although the higher concentration of the cubic phase may reduce the physical hardness of translucent zirconia compared to traditional zirconia, its hardness score and flexural strength remain superior to those of lithium disilicate ceramic.^[11]^This makes fabrication of monolithic veneer in the laboratory and their handling in clinical settings easier, with minimal risk of breakage during try-in and cementation. The long-term performance veneer fabricated from this translucent zirconia veneer has been verified in the literature.^[12,13]^ The application of opaque liquid on the intaglio surface helped mask the dark color of the abutment while preserving the natural translucency of the outer ceramic layers.

In an attempt to enhance the bonding of zirconia with adhesive cement, various approaches have been introduced. Airborne particle abrasion and tribochemical silica coating, combined with the application of a hydrophobic phosphoric acid monomer, such as 10-methacryloyloxydecyl dihydrogen phosphate (MDP), have shown promising results in laboratory settings, particularly in terms of creating surface irregularities and improving bond strength. These findings suggest that both airborne particle abrasion and tribochemical coating are viable methods for treating zirconia surfaces.^[4]^ However, these methods have inherent drawbacks. The initial bond strength of veneers treated with the tribochemical method is high but may degrade over time due to the hydrolysis of the silica coating.^[14]^ On the other hand, airborne particles can cause microcracks within the material, potentially affecting the long-term performance of the restoration.^[5]^ In comparison to airborne abrasion, acid treatment can be applied uniformly across the entire surface, resulting in a consistent and reliable surface treatment. Studies have shown that acid-etched surfaces offer better shear bond strength and comparable flexural strength compared to those treated with airborne abrasion.^[6,7,15]^ The combination of reinforced micromechanical retention created by acid etching and chemical bonding via 10-MDP-containing bonding agents contributed to the long-term success observed in this case report.

The limitation of using translucent zirconia in this case report is that, although it can improve the esthetic outcome compared to conventional zirconia, it still cannot fully replicate the natural translucency of teeth. The use of acid-etched zirconia veneers is a relatively new approach, and while laboratory results are promising, long-term clinical data remain limited. This case report demonstrated success after a 2-year follow-up, but longer observation periods and larger clinical studies would provide more robust guidance for clinical practice.

## 10. Conclusion

This case report highlights the effective application of zirconia for anterior veneer restoration. The combination of translucent zirconia, a layer of opaque material, and an acid-etched surface technique addressed 2 main drawbacks associated with zirconia: optical translucency and bonding strength. Additionally, by utilizing the physical hardness and opacity of zirconia, it was possible to fabricate the veneer as a monolithic structure, simplifying the laboratory process. The restoration has remained stable after 2 yeasr of function, demonstrating the durability of this treatment modality. The results of this case report suggest that acid-etched translucent zirconia could be a viable option for both esthetic and functional rehabilitation in the anterior region.

## Author contributions

**Conceptualization:** Nguyen Viet Anh, Nguyen Thi Trang, Nguyen Thi Loan.

**Data curation:** Nguyen Viet Anh, Nguyen Thi Trang, Nguyen Thi Loan.

**Formal analysis:** Nguyen Viet Anh, Nguyen Thi Trang, Nguyen Thi Loan.

**Investigation:** Nguyen Viet Anh, Nguyen Thi Trang, Nguyen Thi Loan.

**Methodology:** Nguyen Viet Anh, Nguyen Thi Trang, Nguyen Thi Loan.

**Project administration:** Nguyen Viet Anh, Nguyen Thi Trang, Nguyen Thi Loan.

**Resources:** Nguyen Viet Anh, Nguyen Thi Trang, Nguyen Thi Loan.

**Software:** Nguyen Viet Anh, Nguyen Thi Trang, Nguyen Thi Loan.

**Supervision:** Nguyen Viet Anh, Nguyen Thi Trang, Nguyen Thi Loan.

**Validation:** Nguyen Viet Anh, Nguyen Thi Trang, Nguyen Thi Loan.

**Visualization:** Nguyen Viet Anh, Nguyen Thi Trang, Nguyen Thi Loan.

**Writing – original draft:** Nguyen Viet Anh, Nguyen Thi Trang, Nguyen Thi Loan.

**Writing – review & editing:** Nguyen Viet Anh, Nguyen Thi Trang, Nguyen Thi Loan.
